# Aetiology of Acute Febrile Episodes in Children Attending Korogwe District Hospital in North-Eastern Tanzania

**DOI:** 10.1371/journal.pone.0104197

**Published:** 2014-08-04

**Authors:** Coline Mahende, Billy Ngasala, John Lusingu, Allvan Butichi, Paminus Lushino, Martha Lemnge, Zul Premji

**Affiliations:** 1 Korogwe Research Laboratory, National Institute for Medical Research, Tanga, Tanzania; 2 Department of Medical Entomology and Parasitology, School of Public Health, Muhimbili University of Health and Allied Sciences, Dar es Salaam, Tanzania; Instituto de Higiene e Medicina Tropical, Portugal

## Abstract

**Introduction:**

Although the burden of malaria in many parts of Tanzania has declined, the proportion of children with fever has not changed. This situation underscores the need to explore the possible causes of febrile episodes in patients presenting with symptoms at the Korogwe District Hospital (KDH).

**Methods:**

A hospital based cross-sectional study was conducted at KDH, north-eastern Tanzania. Patients aged 2 to 59 months presenting at the outpatient department with an acute medical condition and fever (measured axillary temperature ≥37.5°C) were enrolled. Blood samples were examined for malaria parasites, human immunodeficiency virus (HIV) and bacterial infections. A urine culture was performed in selected cases to test for bacterial infection and a chest radiograph was requested if pneumonia was suspected. Diagnosis was based on both clinical and laboratory investigations.

**Results:**

A total of 867 patients with a median age of 15.1 months (Interquartile range 8.6–29.9) were enrolled from January 2013 to October 2013. Respiratory tract infections were the leading clinical diagnosis with 406/867 (46.8%) of patients diagnosed with upper respiratory tract infection and 130/867 (15.0%) with pneumonia. Gastroenteritis was diagnosed in 184/867 (21.2%) of patients. Malaria infection was confirmed in 72/867 (8.3%) of patients. Bacterial infection in blood and urine accounted for 26/808 (3.2%) infections in the former, and 66/373 (17.7%) infections in the latter. HIV infection was confirmed in 10/824 (1.2%) of patients. Respiratory tract infections and gastroenteritis were frequent in patients under 36 months of age (87.3% and 91.3% respectively). Co-infections were seen in 221/867 (25.5%) of patients. The cause of fever was not identified in 65/867 (7.5%) of these patients.

**Conclusions:**

The different proportions of infections found among febrile children reflect the causes of fever in the study area. These findings indicate the need to optimise patient management by developing malaria and non-malaria febrile illnesses management protocols.

## Introduction

More than half of the children presenting at health facilities in Africa are estimated not to have a malaria infection [1], [2]. The lack of knowledge on the prevalence of the causative agents of other febrile illnesses, the limited access to affordable diagnostic tests as well as the fear of overlooking a life threatening malaria infection, result in most febrile cases being treated and managed as malaria [3], [4].

Fever commonly accompanies a wide range of childhood illnesses and if the patient is not properly managed, this can have potentially serious outcomes including death [5]. In malaria endemic countries, febrile episodes are commonly associated with malaria infection [6], [7]. As current epidemiological data indicates a decline in malaria infections in many parts of sub Saharan Africa, including Tanzania, other causative agents of febrile illnesses should be investigated [8], [9].

Acute febrile episodes are caused by various pathogenic organisms (such as viruses and bacteria), and infections with these agents result in patients presenting with clinical symptoms which resemble those of a malaria infection [10]. An accurate clinical diagnosis without laboratory confirmation can be difficult to make and misleading [11], [12]. Despite efforts by the World Health Organisation (WHO) to improve the diagnosis of malaria through its updated integrated management of childhood illness (IMCI) guidelines, confirmatory diagnosis of malaria is still not adequately carried out. In routine practice, clinicians have continued prescribing antimalarial drugs to malaria negative patients [13], [14]. As IMCI approach is apparently not specific on the diagnosis of non-malarial febrile illnesses [15]. This results in the inappropriate use of antimalarial drugs, possible development of antimicrobial drug resistance and also elevates treatment costs due to unnecessary prescriptions. Incorrect prescriptions may leave patients untreated, thereby unnecessarily increasing morbidity and mortality in these resource poor settings [16].

There is limited data on the epidemiological causes of febrile illnesses (other than malaria) among outpatient children, as well as a lack of reliable surveillance data in Tanzania. A recent study conducted in Tanzania found that malarial infections were no longer a major cause of fever among outpatient children [17]. Disease-specific studies among febrile patients have been conducted in few settings across Sub Saharan Africa. These studies focused mainly on malaria, bacteraemia and human immunodeficiency virus (HIV) infection among inpatients [18]–[20].

Information on the prevalence of local infections, that is based on diagnosis using both clinical presentation and laboratory confirmatory tests is critical for correct management of both malarial and non-malarial febrile illnesses. The main objective of this study was to describe the common causes of fever in children presenting at an outpatient department at the Korogwe District Hospital, north-eastern Tanzania.

## Methods

### Study area

The study was conducted at Korogwe District Hospital (KDH) in Korogwe District, Tanga Region, north-eastern part of Tanzania. The Korogwe District has a population of approximately 73,275 children under the age of five years according to the Tanzanian population and housing census of 2012 [21]. The District has a population growth rate of 2.7% per annum [21]. The environment is characterised by daily temperatures varying from 18°C to 20°C during the rainy season and 26°C to 30°C during the dry season. The annual rainfall ranges from 700–1000 mm with long rainy seasons extending from March to May. The majority of the inhabitants reside in rural settings, practicing subsistence farming and informal trade. The hospital receives 6000 (2010 estimates) children under the age of five years as outpatients annually. The most common clinical diagnoses among children under the age of five years have been malaria, pneumonia, gastroenteritis, septicaemia, anaemia and diarrhoea (Korogwe District Medical Officer, personal communication).

The prevalence of *Plasmodium falciparum* malaria parasitaemia from the community in lowland villages has decreased from 78% in 2003 to 13% in 2008, whereas in the highland villages, the decrease was from 25% to 3% during the same time period [22]. The prevalence of HIV infection among women attending the antenatal clinic at KDH was 2.5% for 2010 [23]. Pneumococcal and rotavirus vaccines were introduced into Tanzania in January 2013 as part of the Expanded Program on Immunization. Vaccine coverage for other vaccines in the Expanded Program on Immunization (EPI) has been above 80%. These vaccines include; Bacille Calmette–Guérin, pentavalent vaccine (diphtheria–tetanus–pertussis, hepatitis B and haemophilus influenza type B), poliovirus and measles vaccines.

### Study population

Sick children aged between 2 and 59 months presenting at KDH outpatient department were assessed for study eligibility from January 2013 to October 2013. Enrolment took place Mondays to Fridays every week. The inclusion criteria were: children aged 2 to 59 months of age presenting at KDH with an acute medical condition and a history of fever in the last 48 hours or measured axillary temperature of ≥37.5°C. The visit should be their first consultation for the present problem. Patients younger than 2 months of age were excluded in the study. Patients were excluded if they had planned admissions (e.g. elective surgery), trauma/injury, if they required an emergency intervention or if they had taken antimalarial drugs and/or antibiotics within the last seven days.

### Ethics statement

The study obtained ethical clearance with reference number NIMR/HQ/R.8a/Vol.1X/1373 from the Tanzanian Medical Research Coordinating Committee. A written informed consent form was issued and signed by the parent/legal guardian of every child enrolled in the study.

### Study procedure

A detailed medical history and a thorough clinical examination were performed on each patient and this information was recorded on a standardised Case Report Form (CRF). This information included: demographic information, clinical history and physical examination data (including weight, axillary temperature and respiratory rate). A chest radiograph was requested if pneumonia was suspected. A clinical diagnosis was made based on the presenting signs and symptoms according to the IMCI guidelines [24]. This ensured that patients could be appropriately managed according to national standard practice while laboratory tests were being processed. Patients who had positive culture results were contacted and the necessary required treatment provided. The Final diagnosis was based on both the clinical presentation and the laboratory findings for malaria microscopy, bacteriology, radiology and HIV testing.

### Laboratory investigations

A maximum of 5 mL venous blood for laboratory investigations was drawn from every patient once inclusion criteria had been met. Each blood draw was conducted aseptically in order to avoid contamination. The venipuncture site was disinfected with 70% isopropyl alcohol and allowed to dry prior to blood drawing.

### Diagnosis of Plasmodium species and *Borrelia* parasitic infection

Thick and thin blood smears were prepared (in duplicate) from the blood collected in the ethylene diamine tetra acetic acid (EDTA) tubes. The thin film was fixed with methanol and blood slides were stained with a 5% Giemsa solution for identification and quantitation of asexual *Plasmodium falciparum* and other *Plasmodium* species. The blood slides were read by two expert microscopists and in case of discrepancy, a third reading was performed. Asexual parasites were counted against 200 (500 if parasite count was <10) white blood cells. A blood slide was considered negative for *Plasmodium* species if no parasites were seen in at least 100 oil-immersion high power fields on the thick film. During examination of the slides for *Plasmodium* species, *Borrelia* species were also screened. *Borrelia* species are seen as spirochaetes under microscopy.

### Differential complete blood count

Blood collected in anticoagulated EDTA tubes was analysed for differential complete blood count using automated MS4s haematology analyser (Melet Schloesing Diamond Diagnostics, USA).

### Aerobic blood culturing

Blood collected in commercially available BD BACTEC PEDS PLUS culture bottles were incubated in an automated blood culture system BACTEC 9050 (Becton Dickinson, Sparks, Maryland, USA). A single blood culture bottle was used per patient. The minimal volume inoculated was 2 mL (3 mL was an ideal volume) and blood culture bottles were incubated for a maximum of five days (unless they flag positive). The BACTEC 9050 detects positive cultures based on CO_2_ production. Blood culture bottles which flagged positive were cultured on standard media with the use of routine microbiological techniques. Analytical Profile Index (API) biochemical test kit (BioMérieux, France) and/or serological tests were used to confirm suspected pathogens.

### Definition of positive and negative culture

A blood culture was considered positive when: a definite pathogen was isolated (e.g. *Streptococcus pneumoniae*, *Streptococcus agalactiae*, *Streptococcus pyogenes*, *Haemophilus influenzae*, *Salmonella* species), a bacteria that could be either a pathogen or a contaminant was isolated within 48 hours of blood culture incubation (e.g. *Escherichia coli*, *Klebsiella pneumoniae*, *Staphylococcus aureus*, *Enterococcus faecalis* group D). Blood cultures were considered to be negative if there were no bacteria isolated after five days incubation.

### Definition of contamination

A blood culture was considered to be contaminated if one or more of the following organisms were identified; coagulase-negative *Staphylococcus* species, *Corynebacterium* species, *alpha- or gamma-hemolytic streptococci, Micrococcus* species, *Bacillus* species and *Propionibacterium* species. However, a definitive diagnosis of a contaminant was decided by a clinician based on the clinical presentation of the patient due to the fact that these organisms could be the cause of an opportunistic infection.

### Urine culturing

Urine was collected from patients presenting with fever, dysuria and/or no obvious cause of fever on clinical examination. A midstream urine was collected in a sterile container. The perineum was swabbed with chlorhexidine and the sample was collected using a sterile urine collector for the younger patients who were not able to follow instructions. Urinalysis by dipstick (URiScan, YD Diagnostics, Korea) was used for presumptive diagnosis of urinary tract infections for early initiation of therapy while urine cultures were being processed. The urine was cultured by inoculation onto cysteine lactose electrolyte deficient agar, MacConkey agar and sheep blood agar. An API biochemical test kit (BioMérieux, France) was used to confirm suspected pathogens. A definitive diagnosis of urinary tract infection was based on the isolation of a bacteria pathogen from the positive culture containing >10^5^ colony-forming units (CFU)/ml [25].

### Antimicrobial susceptibility testing

The Kirby-Bauer disc diffusion method was used for in-vitro antimicrobial susceptibility testing for all pathogenic bacterial isolates from blood and urine cultures. The testing was performed on the following antimicrobial agents; amoxicillin, chloramphenicol, ceftriaxone, ciprofloxacin, co-trimoxazole, gentamicin and penicillin. Reading and interpretation of zone sizes was performed using criteria stipulated by the Clinical and Laboratory Standards Institute (CLSI).

### Human immunodeficiency virus (HIV) testing

HIV antibody testing was performed after patients were informed and counselled as per the National Health Guidelines. Blood drawn from patients was tested for the presence of HIV-1 and HIV-2 antibodies according to the National HIV rapid testing algorithm using the Determine HIV-1/2 Assay Kit (Abbott Laboratories, Abbott Park, IL, USA) [26]. If this was positive, confirmation was carried out using the Uni-Gold HIV (Trinity Biotech, Bray, Co Wicklow, Ireland). For children under the age of 18 months, HIV testing was performed using HIV-1 RNA PCR.

### Case definitions

Fever was defined as history of abnormally high body temperature as reported by the parent/guardian and/or measured axillary temperature ≥37.5°C on presentation. Malaria infection was defined as fever with the presence of asexual *Plasmodium falciparum* parasites in a blood smear confirmed by microscopy. Bacteremia was defined as fever with isolation of pathogenic bacteria from blood culture. Anaemia was defined as haemoglobin concentration below 9.3 g/dL. Clinical diagnoses of respiratory infections and gastroenteritis were defined according to the IMCI guidelines [24].

### Data management and statistical analysis

Data management involved double entry and validation using Microsoft Access 2007. Data analysis was done by STATA version 11.2 (Stata Corp LP, College Station, Texas, USA). Variables were summarized as frequencies and proportions and medians and inter-quartile ranges, as appropriate. Data was compared using the chi-square (*X^2^*) test when comparing proportions. Odds ratios (OR) and 95% confidence intervals (95% CI) were calculated as appropriate. The value of *p*<0.05 was considered statistically significant.

## Results

### Demographic characteristics of the study population

**Figure 1:**
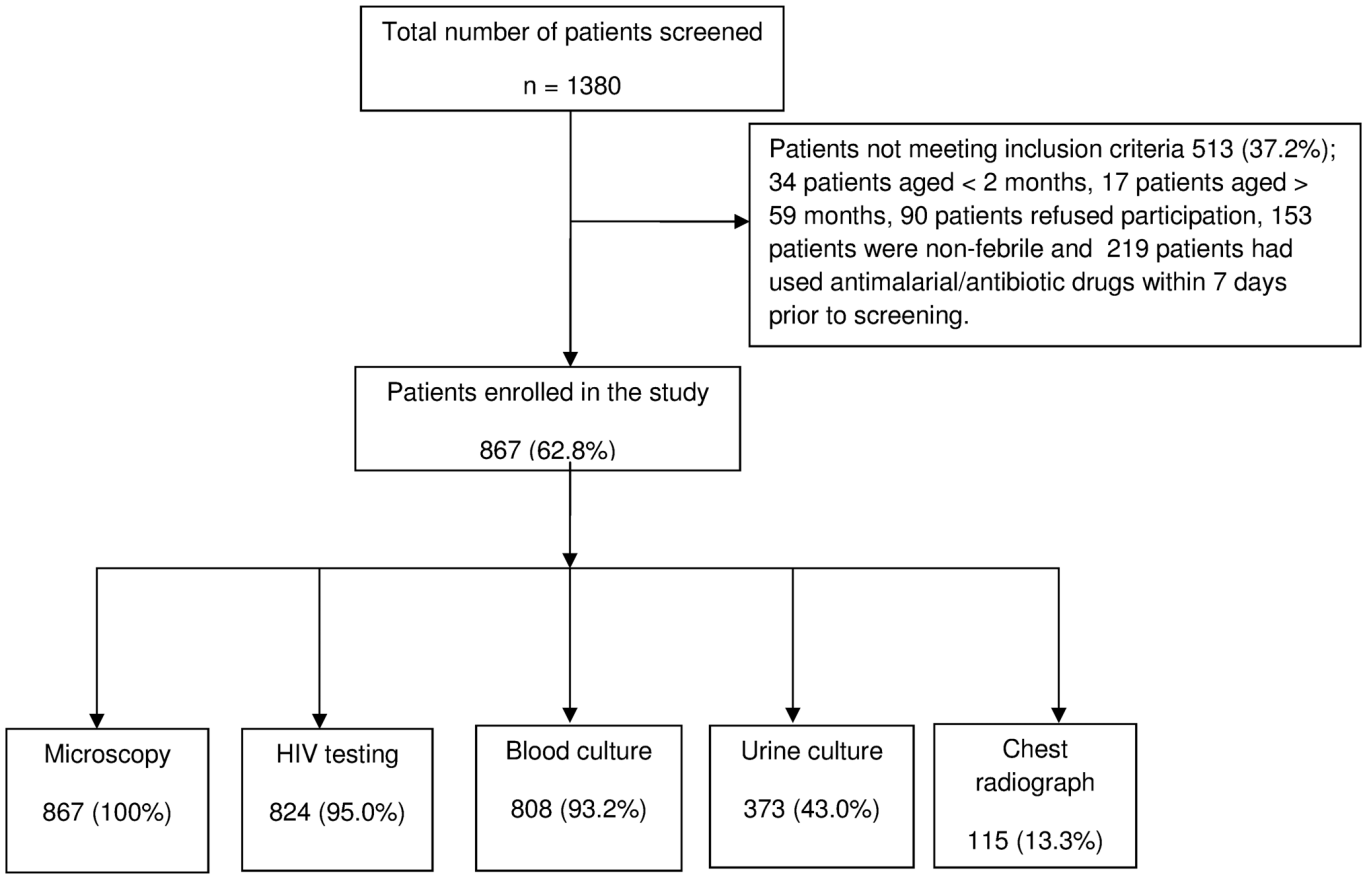
Study flow diagram of patient enrollment at KDH and the investigations performed.

A total of 1380 patients attending the outpatient department at KDH from January 2013 to October 2013 were screened. Of these, 867/1380 (62.8%) were febrile children who were eligible for enrolment (Figure 1). The median age was 15.1 months (Interquartile range (IQR): 8.6–29.9) and girls comprised of 459/867 (52.9%) patients as shown on Table 1. Median axillary body temperature was 38.1°C (IQR 37.5–38.7). The common presenting clinical symptoms other than fever were cough 411/867 (47.4%) and diarrhoea 174/867 (20.1%).

**Table 1:** Demographic characteristics, clinical diagnosis and laboratory findings of patients enrolled at KDH.

Patient characteristics	n, n/N	Proportions (%)
**Gender**		
Girls	408	47.1
**Age**		
2–11 months	356	41.1
12–35 months	370	42.7
36–59 months	141	16.3
**Fever**		
Duration of fever in days, median (IQR)	2 (1–2)	-
Axillary temperature ≥37.5°C	627	72.3
**Common symptoms**		
Cough	411	47.4
Diarrhoea	174	20.1
Difficulty breathing	97	11.2
**Clinical diagnosis**		
Respiratory infections		
Upper respiratory tract infection	406/867	46.8
Pneumonia	130/867	15
Pulmonary Tuberculosis	1/867	0.1
Asthma	19/867	2.2
Gastroenteritis	184/867	21.2
Dysentery	6/867	0.7
Otitis media	5/867	0.6
Fungal infections	6/867	0.7
Skin infections/soft tissue infections	33/867	3.8
Other infections	44/867	5.1
**Laboratory findings**		
*Plasmodium falciparum*	72/867	8.3
Geometric mean parasitemia (range)	37635 (159–1656400)	-
Human immunodeficiency virus	10/824	1.2
Anaemia	226/841	26.9
**Blood culture positive**	26/808	3.2
Non-typhi salmonella	2/26	7.7
*Salmonella typhi*	17/26	65.4
*Streptococcus pneumoniae*	4/26	15.4
Other†	3/26	11.5
**Urine culture**	66/373	17.7
*Escherichia coli*	37/66	56.1
*Klebsiella pneumoniae*	7/66	10.6
*Proteus mirabilis*	5/66	7.6
Other‡	17/66	25.7
**Pyrexia of Unknown Origin**	65/867	7.5

† *Escherichia coli* (1), *Enterobacter cloacae* (1), *Staphylococcus* aureus (1).

‡ *Acinetobacter baumanii* (1), *Citrobacter koseri* (1), *Pseudomonas aeruginosa* (2), *Staphylococcus aureus* (6) *Staphylococcus saprophyticus* (3), *Streptococcus faecalis* (1), *Streptococcus mitis* (1), *Streptococcus viridans* (2).

### Malaria infection

Malaria infection due to *Plasmodium falciparum* was confirmed in 72/867 (8.3%, 95%CI, 6.5–10.1) of the patients identified by thick and thin blood smears. Anaemia was found in 226/841 (26.9%) patients, of whom 36/226 (15.9%) had malaria which increased the risk of anaemia (OR: 3.6 [95%CI: 2.1–6.1] p<0.01). No other *Plasmodia* species or *Borrelia* species were found. Overlap of clinical symptoms was observed among patients with malaria infection; 14/72 (19.4%) and 4/72 (5.6%) patients had clinical symptoms of respiratory tract infections and gastroenteritis respectively.

### Bacteremia

Blood cultures were collected and analysed from 808 patients, of which 26/808 (3.2%, 95%CI, 1.9–4.4) were positive for pathogenic bacterial growth. *Salmonella typhi* was the predominant bacteria isolated in 17/26 (65.4%) of cases followed by *Streptococcus pneumoniae* 4/26 (15.4%) as shown in Table 1.

### Urinary tract infection

A Urinary tract infection was confirmed in 66/373 (17.7%, 95%CI, 14.0–22.0) of patients and there was no significant association with gender (36 boys, 30 girls, *X^2^* = 0.074, P = 0.786). The most commonly isolated bacteria were *Escherichia coli* 37/66 (56.1%) followed by *Klebsiella pneumoniae* 7/66 (10.6%), *Staphylococcus aureus* 6/66 (9.1%) and *Proteus mirabilis* 5/66 (7.6%).

### HIV infection

HIV testing was performed in 824/867 (95.0%) patients and among them, 10/824 (1.2%, 95%CI, 0.6–2.2) were confirmed positive for infection with HIV. None of the HIV infected patients had either a malarial or a bacterial infection.

### Clinical diagnoses

#### Respiratory tract infections

Upper respiratory tract infections were the most common clinical diagnoses presenting in 406/867 (46.8%, 95%CI, 43.5–50.2) of patients. Pneumonia was clinically diagnosed in 130/867 (15.0%, 95%CI, 12.7–17.5) of patients. Pneumonia confirmed by chest radiograph was accounted in 54/130 (41.5%) of these cases and five patients had diagnosis of severe pneumonia. *Salmonella typhi* (n = 2) and *Escherichia coli* (n = 1) were isolated from single blood cultures of patients with upper respiratory tract infections. *Streptococcus pneumoniae* (n = 1) and *Staphylococcus aureus* (n = 1) were isolated from single blood cultures of patients with pneumonia diagnosis. One patient was suspected to have pulmonary tuberculosis from clinical symptoms and chest radiograph results.

#### Gastroenteritis

Gastroenteritis was diagnosed in 184/867 (21.2%, 95%CI, 18.5–24.1) of patients. Dysentery was diagnosed in 6 patients. *Salmonella typhi* (n = 6) and non-typhi salmonella (n = 1) were isolated from the blood cultures of patients with gastroenteritis.

#### Other infections

Other infections are summarised on Table 1. The cause of fever could not be identified in 65/867 (7.5%, 95%CI, 5.8–9.5) of the patients.

#### Distribution of diagnoses according to age

**Figure 2:**
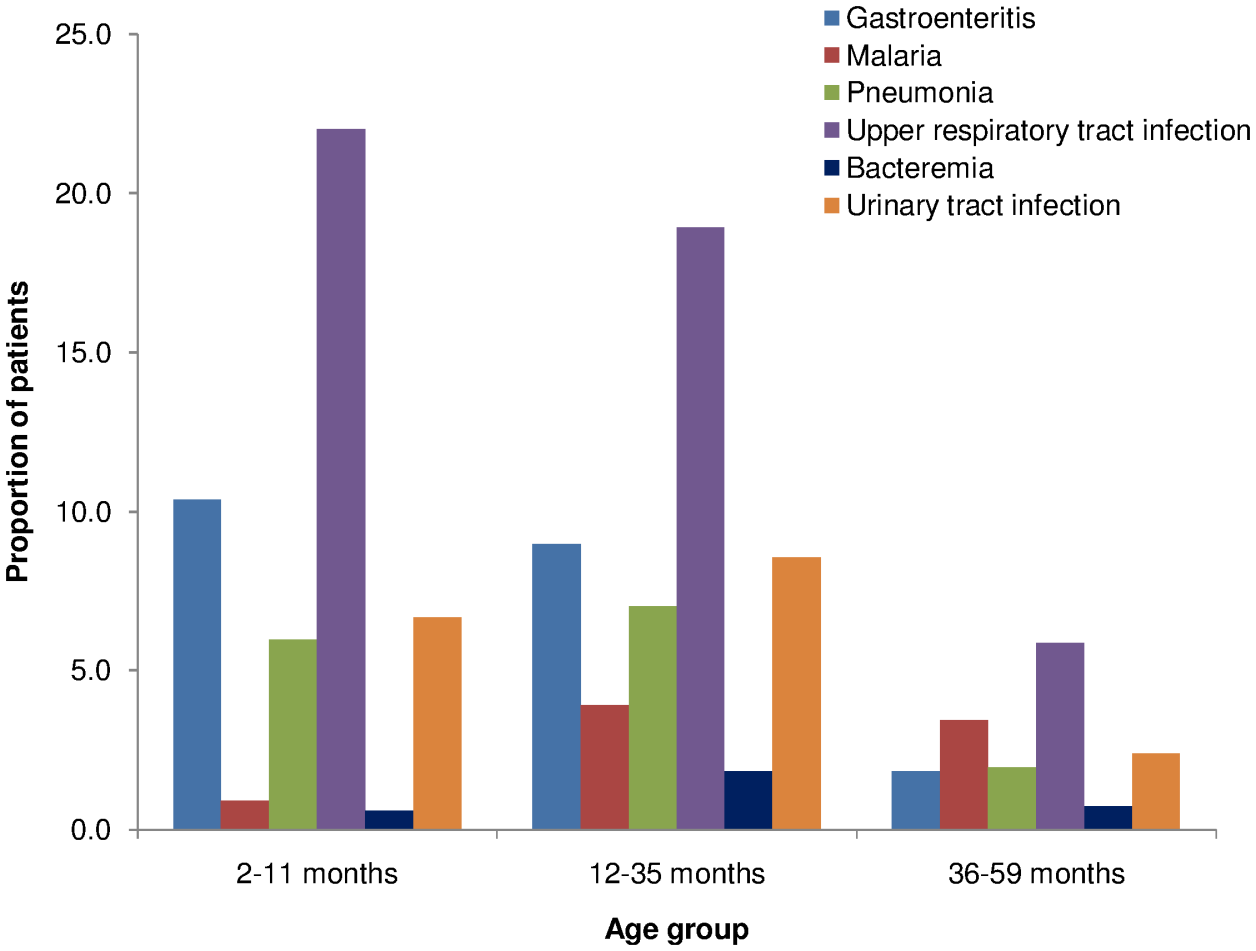
Distribution of common diagnosis according to age group.

The majority of the patients under the age of 36 months presented with respiratory tract infections 468/536 (87.3%). The second most common diagnosis was gastroenteritis 168/184 (91.3%) and thirdly, urinary tract infections 57/66 (86.4%). Malaria infection 8/72 (11.1%) was less common in patients under the age of 12 months (Figure 2).

### Diagnosis of hospitalised patients

**Figure 3:**
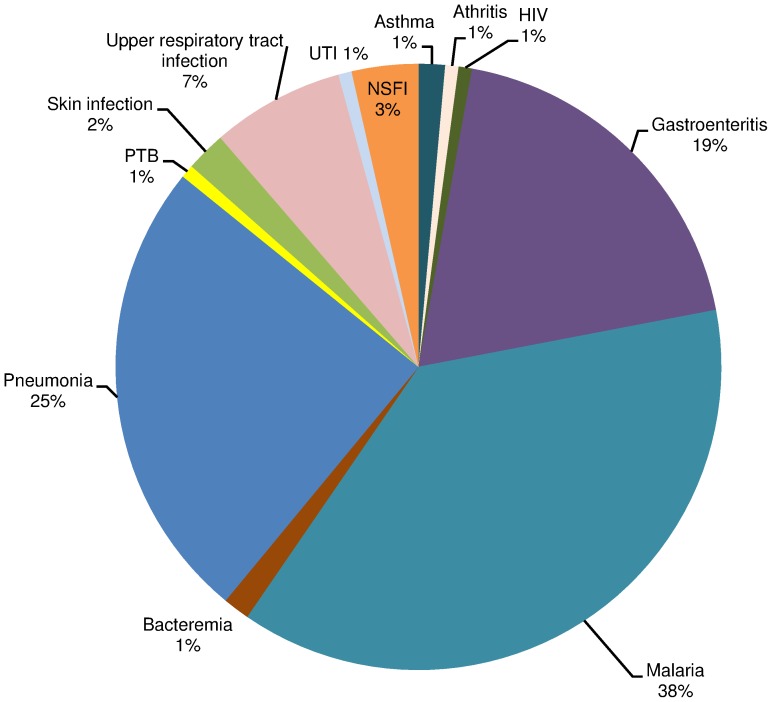
Distribution of illnesses among hospitalised patients at KDH (n = 141).

Following enrolment, 141/867 (16.3%) patients were hospitalised, of whom 2/141 (1.4%) died from severe pneumonia and upper respiratory tract infection. Malaria infection was a leading cause of admission with 53/141 (37.6%) of patients, followed by pneumonia 35/141 (24.8%), gastroenteritis 27/141 (19.1%) and other infections as indicated in Figure 3.

### Co- infections

**Figure 4:**
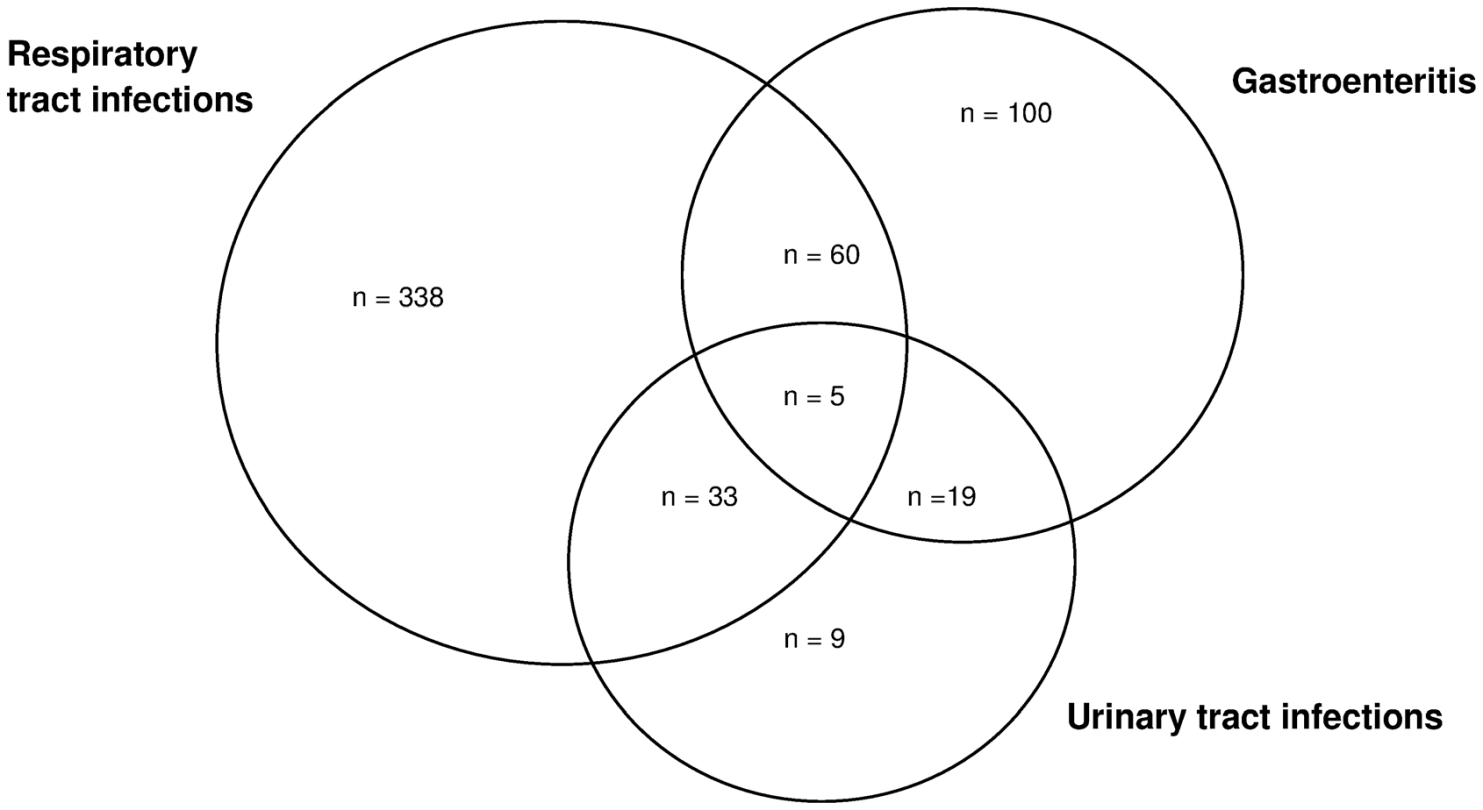
Co-infections of common diagnosis.

Co-infections were common and were observed in 221/867 (25.5%, 95%CI, 22.6–28.5) of the patients. Urinary tract infections were present in 33/436 (7.6%) patients with respiratory tract infections and in 19/184 (10.3%) patients with gastroenteritis (Figure 4). Five patients had multiple diagnoses of urinary tract infection, respiratory tract infection and gastroenteritis. Among patients with malaria, 8/72 (11.1%) patients had urinary tract infections. Sixty patients had both respiratory tract infections as well as gastroenteritis.

### Antimicrobial susceptibility pattern

Gram negative bacterial isolates demonstrated good susceptibility to ciprofloxacin (80%–100%), moderate susceptibility to gentamicin (50%–72%) and poor susceptibility to amoxicillin, chloramphenicol and co-trimoxazole (3%–14%). *Escherichia coli* and *Klebsiella pneumoniae* demonstrated poor susceptibility to ceftriaxone (35.1% and 14.3% respectively). *Klebsiella pneumoniae* was the only Gram negative bacteria with good susceptibility to chloramphenicol (71.4%). The Gram positive bacterial isolates were observed to have good susceptibility to ceftriaxone and chloramphenicol (75%–100%) and poor susceptibility to co-trimoxazole and penicillin (0%–50%).

## Discussion

This study has been able to contribute information on the prevalence of the possible causes of common febrile episodes among children less than five years of age, attending an outpatient department at KDH in north-eastern Tanzania. Over three quarters of children presenting with fever were under the age of 36 months and the majority had respiratory tract infections and gastroenteritis as the leading clinical diagnosis. Younger children under the age of 36 months tend to be more vulnerable to infections due to their immature immunity, different modes of exposure to pathogenic organisms and possibly due to a low rate of immunisation [27], [28].

*Plasmodium falciparum* malaria, invasive community-acquired bacteria and HIV infections were uncommon among febrile children in the study with a prevalence of 8.3%, 3.2% and 1.2%, respectively. These findings are consistent with other studies on the declining malaria and community-acquired bacterial infections in the region [29], [30]. The low malaria prevalence rate might partly be due to current interventions which include use of insecticide treated nets, the wide availability of effective antimalarial drugs, residual spraying and change of malaria vector behaviour [31], [32]. This underscores the importance of the rational use of antibiotic and antimalarial drugs in evidence based medicine among less severely ill patients in order to counteract the problem of drug resistance.

This study found an increasing prevalence of *Salmonella typhi* among young children compared with that of non-typhi salmonella. These findings are in keeping with those of Biggs and colleagues, who found similar changing pattern of bacterial infections where *Salmonella typhi* compared with non-typhi salmonella was more common in lower malaria prevalent areas [20]. The observed prevalence of *Salmonella typhi* indicates the presence of typhoid fever among these young rural patients. The following critical measures can be implemented in preventing typhoid fever in this resource poor area through improving the quality of life. This can be done by ensuring good sanitation, safe water supply and public health education on personal as well as food hygiene.

Amoxicillin, chloramphenicol and co-trimoxazole are the most widely available antimicrobial agents in the community. These were shown to demonstrate poor susceptibility to the leading Gram negative bacterial isolates from both blood and urine cultures. Ciprofloxacin was effective but not recommended for use in children under the age of five years [33], [34]. The findings support previously published work and emphasize the increasing problem of antimicrobial drug resistance [35], [36]. Specific identification of bacteria causing infections is important for understanding and monitoring of antimicrobial drug resistance patterns. This will ensure patients especially children are provided with appropriate and effective treatment.

The prevalence of urinary tract infections in this study was higher among children under 36 months of age and these patients were often co-infected. The lack of access to clean water and good sanitation in this rural community might be the reason for the higher prevalence of infections observed. As in most studies conducted on urinary tract infections, *Escherichia coli* were found to be the predominant isolate. This study did not find any significant difference in gender among children with urinary tract infection. The burden of urinary tract infections in young children living in developing countries remains undefined, despite being the most common bacterial infections in childhood. The diagnosis of urinary tract infection is important in young children since if left untreated can cause nephrourologic abnormalities and impaired renal function in future [37]. With an increase in the prevalence of urinary tract infections among young children, findings from this study recommend routine urine testing of febrile children from rural communities where living conditions are poor [17], [36].

Half of the febrile children in the study were clinically diagnosed with respiratory tract infections and nearly a quarter had gastroenteritis making these the leading clinical diagnoses. The observations were more frequent in children under the age of 36 months. The majority of respiratory tract infections and diarrhoea in children are known to be of viral origin [38]–[40], [40]–[42]. A recent study conducted in Tanzanian children has indicated nearly half of acute respiratory infections were caused by respiratory viruses [17]. Unfortunately, antibiotics are routinely and continuously used to treat these viral infections, contributing to the increase burden of antimicrobial drug resistance. The current study provides preliminary data for future evidence-based research on respiratory and enteric viruses in the community.

Co-infections were observed in a quarter of the patients, mostly among those with urinary tract infections, respiratory tract infections and gastroenteritis. Existence of multiple infections is a challenging issue when developing fever management guidelines, especially in poor rural areas which are usually at higher risks of numerous kinds of infections [43]. This study has underlined the prevalence of co-infections that exist among patients enrolled in the study area.

The causative agents of febrile infections documented in this study can be considered as representative of the causes of fever in this specific area. Despite recommendations by WHO on the parasitological evidence for malaria treatment, management of fever cases in Tanzania is still challenging [44], [45]. Furthermore, IMCI guidelines remain unspecific for the management of non-malaria febrile illnesses. Evidence based findings on bacterial infections from the current study can contribute to the revision of clinical algorithms for management of febrile illnesses. There is a need for guidelines that would assist in the management of non-malarial febrile illnesses given the declining malaria trend. Febrile illnesses, especially in children, require proper management so as to avoid unnecessary morbidity and mortalities, antimicrobial abuse and unnecessary expense. Investment in diagnostic facilities, such as adequate microbiology laboratories, qualified and trained laboratory personnel is needed. Furthermore, availability of rapid diagnostic tests for malaria, bacterial and viral infections, is required for rapid and accurate diagnoses of paediatric infections. A possible recommendation is that fever surveillance be conducted along side the existing malaria surveillance systems in future clinical research on vaccines and drugs.

The study had a number of limitations. Firstly, the study was conducted for the period of ten months and therefore could not assess seasonality of febrile episodes. Secondly, the study relied on verbal information provided by parents and guardians on the prior use of antimalarial drugs and antibiotics. This means that some of the children enrolled may well have been on antimicrobial therapy and this will have contributed to the low yield of positive cultures. In addition, the collection of only a single blood culture is known to reduce the possibility of isolating the organisms present in the bloodstream. The volume of blood inoculated into culture bottles was sometimes low due to difficulty in getting venous access especially from young patients and this might have contributed to the low bacterial yield. The study could not provide evidence-based data on respiratory and enteric viruses which are thought to cause majority of respiratory tract infections and diarrhoea in young children. Causative agents of zoonosis among outpatient children from this particular study community were not investigated. Understanding their prevalence would be of advantage despite not being investigated routinely at health facilities in the country.

## References

[1] Gething PW, Kirui VC, Alegana VA, Okiro EA, Noor AM, et al (2010) Estimating the number of paediatric fevers associated with malaria infection presenting to Africa's public health sector in 2007. PLoS Med 7: e1000301 10.1371/journal.pmed.1000301 20625548 PMC2897768

[2] Osei-Kwakye K, Asante KP, Mahama E, Apanga S, Owusu R, et al (2013) The benefits or otherwise of managing malaria cases with or without laboratory diagnosis: the experience in a district hospital in Ghana. PLoS One 8: e58107 10.1371/journal.pone.0058107 23505457 PMC3591456

[3] Archibald LK, Reller LB (2001) Clinical microbiology in developing countries. Emerg Infect Dis 7: 302–305 10.3201/eid0702.700302 11294729 PMC2631738

[4] Gwer S, Newton CR, Berkley JA (2007) Over-diagnosis and co-morbidity of severe malaria in African children: a guide for clinicians. Am J Trop Med Hyg 77: 6–13 77/6_Suppl/6 [pii] 18165469 PMC2669774

[5] Petit PL, van Ginneken JK (1995) Analysis of hospital records in four African countries, 1975–1990, with emphasis on infectious diseases. J Trop Med Hyg 98: 217–227. 7636917

[6] Ye Y, Madise N, Ndugwa R, Ochola S, Snow RW (2009) Fever treatment in the absence of malaria transmission in an urban informal settlement in Nairobi, Kenya. Malar J 8: 160 10.1186/1475-2875-8-160 19604369 PMC2717114

[7] Hertz JT, Munishi OM, Sharp JP, Reddy EA, Crump JA (2013) Comparing actual and perceived causes of fever among community members in a low malaria transmission setting in northern Tanzania. Trop Med Int Health 18: 1406–1415 10.1111/tmi.12191 24103083 PMC3943636

[8] D'Acremont V, Lengeler C, Genton B (2010) Reduction in the proportion of fevers associated with Plasmodium falciparum parasitaemia in Africa: a systematic review. Malar J 9: 240 10.1186/1475-2875-9-240 20727214 PMC2936918

[9] Rutta AS, Francis F, Mmbando BP, Ishengoma DS, Sembuche SH, et al (2012) Using community-owned resource persons to provide early diagnosis and treatment and estimate malaria burden at community level in north-eastern Tanzania. Malar J 11: 152 10.1186/1475-2875-11-152 22554149 PMC3517357

[10] Were T, Davenport GC, Hittner JB, Ouma C, Vulule JM, et al (2011) Bacteremia in Kenyan children presenting with malaria. J Clin Microbiol 49: 671–676 10.1128/JCM.01864-10 21106789 PMC3043473

[11] Petti CA, Polage CR, Quinn TC, Ronald AR, Sande MA (2006) Laboratory medicine in Africa: a barrier to effective health care. Clin Infect Dis 42: 377–382 10.1086/499363 16392084

[12] Urdea M, Penny LA, Olmsted SS, Giovanni MY, Kaspar P, et al (2006) Requirements for high impact diagnostics in the developing world. Nature 444 Suppl 1: 73–79 10.1038/nature05448 17159896

[13] Strom GE, Haanshuus CG, Fataki M, Langeland N, Blomberg B (2013) Challenges in diagnosing paediatric malaria in Dar es Salaam, Tanzania. Malar J 12: 228 10.1186/1475-2875-12-228 23822515 PMC3703277

[14] Moon AM, Biggs HM, Rubach MP, Crump JA, Maro VP, et al (2014) Evaluation of In-Hospital Management for Febrile Illness in Northern Tanzania before and after 2010 World Health Organization Guidelines for the Treatment of Malaria. PLoS One 9: e89814 10.1371/journal.pone.0089814; PONE-D-13-35569 [pii] 24587056 PMC3933647

[15] World Health Organisation (WHO) (2010) Guidelines for the treatment of malaria, 2nd edition.

[16] Reyburn H, Mbatia R, Drakeley C, Carneiro I, Mwakasungula E, et al (2004) Overdiagnosis of malaria in patients with severe febrile illness in Tanzania: a prospective study. BMJ 329: 1212 10.1136/bmj.38251.658229.55 15542534 PMC529364

[17] D'Acremont V, Kilowoko M, Kyungu E, Philipina S, Sangu W, et al (2014) Beyond malaria–causes of fever in outpatient Tanzanian children. N Engl J Med 370: 809–817 10.1056/NEJMoa1214482 24571753

[18] Nadjm B, Amos B, Mtove G, Ostermann J, Chonya S, et al (2010) WHO guidelines for antimicrobial treatment in children admitted to hospital in an area of intense Plasmodium falciparum transmission: prospective study. BMJ 340: c1350. 20354024 10.1136/bmj.c1350 PMC2847687

[19] Crump JA, Ramadhani HO, Morrissey AB, Msuya LJ, Yang LY, et al (2011) Invasive bacterial and fungal infections among hospitalized HIV-infected and HIV-uninfected children and infants in northern Tanzania. Trop Med Int Health 16: 830–837 10.1111/j.1365-3156.2011.02774.x 21470347 PMC3227789

[20] Biggs HM, Lester R, Nadjm B, Mtove G, Todd JE, et al (2014) Invasive salmonella infections in areas of high and low malaria transmission intensity in Tanzania. Clin Infect Dis 58: 638–647 10.1093/cid/cit798 24336909 PMC3922215

[21] National Bureau of Statistics Ministry of Finance Tanzania (2013) 2012 Population and housing census.

[22] Mmbando BP, Vestergaard LS, Kitua AY, Lemnge MM, Theander TG, et al (2010) A progressive declining in the burden of malaria in north-eastern Tanzania. Malar J 9: 216 10.1186/1475-2875-9-216 20650014 PMC2920289

[23] Schmiegelow C, Minja D, Oesterholt M, Pehrson C, Suhrs HE, et al (2013) Malaria and fetal growth alterations in the 3(rd) trimester of pregnancy: a longitudinal ultrasound study. PLoS One 8: e53794 10.1371/journal.pone.0053794; PONE-D-12-29446 [pii] 23326508 PMC3543265

[24] World Health Organisation (WHO) (2005) Intergrated Managemnet of Childhood Illness.

[25] Kwon JH, Fausone MK, Du H, Robicsek A, Peterson LR (2012) Impact of laboratory-reported urine culture colony counts on the diagnosis and treatment of urinary tract infection for hospitalized patients. Am J Clin Pathol 137: 778–784 10.1309/AJCP4KVGQZEG1YDM 22523217

[26] National Aids Control Programme Ministry of Health and Social Welfare Tanzania (2012) Guideline for HIV testing.

[27] Liu L, Johnson HL, Cousens S, Perin J, Scott S, et al (2012) Global, regional, and national causes of child mortality: an updated systematic analysis for 2010 with time trends since 2000. Lancet 379: 2151–2161 10.1016/S0140-6736(12)60560-1 22579125

[28] Hamilton JL, John SP (2013) Evaluation of fever in infants and young children. Am Fam Physician 87: 254–260 d10789 [pii]. 23418797

[29] Mmbando BP, Vestergaard LS, Kitua AY, Lemnge MM, Theander TG, et al (2010) A progressive declining in the burden of malaria in north-eastern Tanzania. Malar J 9: 216 10.1186/1475-2875-9-216 20650014 PMC2920289

[30] Mtove G, Amos B, Nadjm B, Hendriksen IC, Dondorp AM, et al (2011) Decreasing incidence of severe malaria and community-acquired bacteraemia among hospitalized children in Muheza, north-eastern Tanzania, 2006–2010. Malar J 10: 320 10.1186/1475-2875-10-320 22029477 PMC3219788

[31] Murray CJ, Rosenfeld LC, Lim SS, Andrews KG, Foreman KJ, et al (2012) Global malaria mortality between 1980 and 2010: a systematic analysis. Lancet 379: 413–431 10.1016/S0140-6736(12)60034-8 22305225

[32] Aregawi MW, Ali AS, Al-mafazy AW, Molteni F, Katikiti S, et al (2011) Reductions in malaria and anaemia case and death burden at hospitals following scale-up of malaria control in Zanzibar, 1999–2008. Malar J 10: 46 10.1186/1475-2875-10-46 21332989 PMC3050777

[33] The United Republic of Tanzania MoHaSW (2007) Standard Treatment Guidelines (STG) and The National Essential Medicines List (NEMLIT) for Mainland Tanzania (Page 6).

[34] Choi SH, Kim EY, Kim YJ (2013) Systemic use of fluoroquinolone in children. Korean J Pediatr 56: 196–201 10.3345/kjp.2013.56.5.196 23741232 PMC3668199

[35] Thriemer K, Ley B, Ame S, von SL, Pak GD, et al (2012) The burden of invasive bacterial infections in Pemba, Zanzibar. PLoS One 7: e30350 10.1371/journal.pone.0030350; PONE-D-11-19140 [pii] 22363426 PMC3281825

[36] Msaki BP, Mshana SE, Hokororo A, Mazigo HD, Morona D (2012) Prevalence and predictors of urinary tract infection and severe malaria among febrile children attending Makongoro health centre in Mwanza city, North-Western Tanzania. Arch Public Health 70: 4 10.1186/0778-7367-70-4 22958592 PMC3415110

[37] Montini G, Tullus K, Hewitt I (2011) Febrile urinary tract infections in children. N Engl J Med 365: 239–250 10.1056/NEJMra1007755 21774712

[38] Feikin DR, Njenga MK, Bigogo G, Aura B, Aol G, et al (2013) Viral and bacterial causes of severe acute respiratory illness among children aged less than 5 years in a high malaria prevalence area of western Kenya, 2007–2010. Pediatr Infect Dis J 32: e14–e19 10.1097/INF.0b013e31826fd39b 22914561

[39] Niang MN, Diop OM, Sarr FD, Goudiaby D, Malou-Sompy H, et al (2010) Viral etiology of respiratory infections in children under 5 years old living in tropical rural areas of Senegal: The EVIRA project. J Med Virol 82: 866–872 10.1002/jmv.21665 20336732 PMC7166331

[40] Bonkoungou IJ, Haukka K, Osterblad M, Hakanen AJ, Traore AS, et al (2013) Bacterial and viral etiology of childhood diarrhea in Ouagadougou, Burkina Faso. BMC Pediatr 13: 36 10.1186/1471-2431-13-36 23506294 PMC3616825

[41] Sire JM, Garin B, Chartier L, Fall NK, Tall A, et al (2013) Community-acquired infectious diarrhoea in children under 5 years of age in Dakar, Senegal. Paediatr Int Child Health 33: 139–144 10.1179/2046905512Y.0000000046 23930725

[42] Moyo SJ, Gro N, Matee MI, Kitundu J, Myrmel H, et al (2011) Age specific aetiological agents of diarrhoea in hospitalized children aged less than five years in Dar es Salaam, Tanzania. BMC Pediatr 11: 19 10.1186/1471-2431-11-19 21345186 PMC3050719

[43] Alemu A, Shiferaw Y, Addis Z, Mathewos B, Birhan W (2013) Effect of malaria on HIV/AIDS transmission and progression. Parasit Vectors 6: 18 10.1186/1756-3305-6-18 23327493 PMC3564906

[44] Mubi M, Kakoko D, Ngasala B, Premji Z, Peterson S, et al (2013) Malaria diagnosis and treatment practices following introduction of rapid diagnostic tests in Kibaha District, Coast Region, Tanzania. Malar J 12: 293 10.1186/1475-2875-12-293 23977904 PMC3765530

[45] Baltzell K, Elfving K, Shakely D, Ali AS, Msellem M, et al (2013) Febrile illness management in children under five years of age: a qualitative pilot study on primary health care workers' practices in Zanzibar. Malar J 12: 37 10.1186/1475-2875-12-37 23356837 PMC3626688

